# Affinity maturation generates greatly improved xyloglucan-specific carbohydrate binding modules

**DOI:** 10.1186/1472-6750-9-92

**Published:** 2009-10-31

**Authors:** Laura von Schantz, Fredrika Gullfot, Sebastian Scheer, Lada Filonova, Lavinia Cicortas Gunnarsson, James E Flint, Geoffrey Daniel, Eva Nordberg-Karlsson, Harry Brumer, Mats Ohlin

**Affiliations:** 1 Dept of Immunotechnology, Lund University, Lund, Sweden; 2 School of Biotechnology, Royal Institute of Technology (KTH), Stockholm, Sweden; 3 Dept of Wood Science, Swedish University of Agricultural Science, Uppsala, Sweden; 4 WURC, Swedish University of Agricultural Science, Uppsala, Sweden; 5 Institute for Cell and Molecular Biosciences, Newcastle University, Newcastle upon Tyne, UK; 6 Dept of Biotechnology, Lund University, Lund, Sweden; 7 Current address: The International Max Plank Research School for Molecular and Cellular Biology, Max-Planck-Institute of Immunobiology, Freiburg, Germany; 8 Current address: Affitech AS, Oslo, Norway

## Abstract

**Background:**

Molecular evolution of carbohydrate binding modules (CBM) is a new approach for the generation of glycan-specific molecular probes. To date, the possibility of performing affinity maturation on CBM has not been investigated. In this study we show that binding characteristics such as affinity can be improved for CBM generated from the CBM4-2 scaffold by using random mutagenesis in combination with phage display technology.

**Results:**

Two modified proteins with greatly improved affinity for xyloglucan, a key polysaccharide abundant in the plant kingdom crucial for providing plant support, were generated. Both improved modules differ from other existing xyloglucan probes by binding to galactose-decorated subunits of xyloglucan. The usefulness of the evolved binders was verified by staining of plant sections, where they performed better than the xyloglucan-binding module from which they had been derived. They discriminated non-fucosylated from fucosylated xyloglucan as shown by their ability to stain only the endosperm, rich in non-fucosylated xyloglucan, but not the integument rich in fucosylated xyloglucan, on tamarind seed sections.

**Conclusion:**

We conclude that affinity maturation of CBM selected from molecular libraries based on the CBM4-2 scaffold is possible and has the potential to generate new analytical tools for detection of plant carbohydrates.

## Background

Plant cell walls rich in polysaccharides are important targets for the food, fiber and fuel industries. Both primary and secondary cell walls consist of a complex network of cellulose microfibrils connected to two different groups of polysaccharides, hemicelluloses and pectins, which together with a lesser amount of glycoproteins and phenolic substances interact to form the plant extracellular matrix. Polysaccharide content varies largely in concentration, type and structure between different plant species, tissues and during the stages of plant development. Less invasive methods that enable analysis of plant components without destroying the network are sought as they can be used not only to detect the presence of individual polysaccharides and their microdistribution across cell walls but can also reveal the organization and interactions between different matrix-components thus helping to understand their function. This is possible with molecular probes that specifically detect polysaccharides in plant sections [1].

To date, antibodies dominate the field of molecular probes but some challenges need still to be overcome. Attempts to produce antibodies by conventional immunization strategies that recognize carbohydrates are often hampered by the low immunogenicity/antigenicity of these macromolecules. Furthermore, antibodies in their native form are unstable under certain conditions like elevated temperatures, and they have a large size that limits their penetration into samples and restricts their use in some applications. These limitations have led to the development and use of techniques that are independent of immunization [2] and to approaches using stabilized protein variants [3]. Furthermore, alternative scaffolds [4] that are stable enough to withstand the modulation of their molecular surface by molecular engineering [5] are used as alternatives to antibodies and antibody fragments to select specific binders from large molecular libraries. Carbohydrate-binding module (CBM) 4-2 from xylanase 10A of *Rhodothermus marinus *is one such scaffold from which a combinatorial library has been constructed through mutagenesis of twelve amino acids in the carbohydrate-binding cleft [6]. From this library, binders with novel engineered specificities targeting carbohydrates have been selected proving the evolutionary capacity of this scaffold.

**Figure 1:**
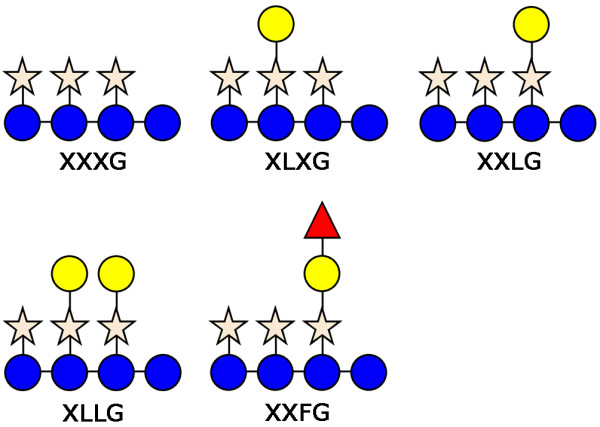
**Xyloglucan building blocks**. Schematic structures of oligosaccharide units XXXG, XLXG, XXLG, XLLG and XXFG that build up xyloglucan of many land plant species. The building blocks are composed of glucosyl units (blue circle) joined together by β1,4 linkages, to which α1,6 linked xylosyl units (orange star) may be added. Galactose (yellow circle) and fucose (red triangle) are incorporated into these structures via β1,2 and α1,2 linkages, respectively.

In this study we further investigated CBM4-2 as a diversity-carrying scaffold and explored the potential of selected variants to undergo further evolution *in vitro *to perfect their binding properties and their usefulness as molecular probes. Random mutagenesis is a powerful tool that can be used to create diversity from which mutants with improved binding can be selected in a manner similar to that exploited by the immune system for adjusting antibody affinity and/or specificity against a given antigen. Here we target the plant polysaccharide xyloglucan. During plant growth, the primary cell wall has a crucial role by providing mechanical support while allowing cell growth and expansion. Of the hemicelluloses present in primary cell walls, xyloglucan is the most abundant in dicotyledons and non-graminaceous monocotyledons. It is built up by a cellulose-like β1-4 glucan backbone that may be substituted up to 75% with xylose units. The xyloses in turn can be decorated with galactose and fucose units, in the latter case generating so-called fucosylated xyloglucan (Figure 1). Many questions still remain to be answered regarding the role of xyloglucan. It is known that xyloglucan structure varies slightly in different plant species [7,8] but its exact composition for all species is unknown. Also the role and necessity of xyloglucan during development of the cellulose-xyloglucan network in primary cell walls is currently under debate [9]. Moreover, recent studies suggest that xyloglucan may play an important role in the bending of hardwood tree by creating tensile stress in the gelatinous G-layer found in the tension wood [10,11]. Molecular probes specific for xyloglucan could help to address these questions in detail.

To date, there exist two types of xyloglucan detecting probes, polyclonal and monoclonal antibodies [12,13], and xyloglucan-binding modules (XGBM) created by CBM engineering [14]. In this study, designed to evaluate the evolutionary potential of CBM by exposing a XGBM to random mutagenesis and selection, we have generated additional xyloglucan binding probes. The new XGBM have higher affinity for xyloglucan compared to our earlier reported XGBM and these bind, in contrast to other xyloglucan detecting probes, to xyloglucan regions that are galactose-decorated, thereby extending the limited set of xyloglucan-specific binders and their usefulness in applications such as studies of plant cell wall organization.

## Methods

### Carbohydrates

Non-fucosylated xyloglucan derived from seeds of *Tamarindus indica*, laminarin, lichenan and galactose were derived from Megazyme (Bray, Ireland) while birchwood xylan was purchased from Sigma-Aldrich (St. Louis, MO, USA). Biotinylated xyloglucan was prepared as previously described [14]. Biotinylated fucosylated xyloglucan was prepared from fucosylated xyloglucan derived from *Rubus fructicosus *(kindly provided by Gérard Chambat, CERMAV-CNRS, Grenoble, France), as previously described [14].

### Library construction

The gene encoding XG-34 [GenBank:DQ279274], a XGBM previously described [14], was used as starting material when creating two xyloglucan focused CBM libraries (hereafter referred to as Lib1 and Lib2). Mutations were randomly introduced by using the PCR-based Genemorph II Random Mutagenisis Kit from Stratagene (La Jolla, CA, USA) and following the manufacture's instructions for achieving a mutation ratio of 1%. In a first round of mutations XG-34 was used as template gene to create Lib1, which in turn was submitted to a second round of mutations to create Lib2. Both PCR-products were purified (from 2% agarose gels) using a commercial kit (QIAquick Gel Extraction Kit, Qiagen, Hilden, Germany), digested with *Sfi*I and *Not*I (both from New England Biolabs, Beverly, MA, USA) and purified again before cloning between *Sfi*I/*Not*I sites of a modified version of the pFab5c.His [6] phagemid vector. The ligated products were transformed into electrocompetent *Escherichia coli *Top10F' (Invitrogen, Carlsbad, CA, USA) that were grown on Luria-Bertani (LB) agar plates (15 cm Ø) containing selective antibiotics (100 μg/ml ampicillin, 10 μg/ml tetracycline and 1% (w/v) glucose). The cultivated cells were gathered and stored at -80°C in 15% glycerol and colonies selected at random for sequence-analysis to determine the diversity of each library.

Phage stocks were produced by infecting cultures of the bacteria harbouring the library grown in 2× Yeast Tryptone (2YT) media containing 100 μg/ml ampicillin, 10 μg/ml tetracycline and 1% (w/v) glucose at exponential growth phase with VSCM13 helper phage (Stratagene) at a multiplicity of infection of 20:1 during 30 min at 37°C without shaking. The growth medium was changed and the glucose was replaced by 0.25 mM isopropyl-β-D-thiogalactoside (IPTG) and 50 μg/ml kanamycin and production of phage particles was allowed to proceed over night at 30°C with shaking. Finally the phages were precipitated by incubation on ice of filtered supernatants with 0.25 volumes 20% PEG6000/2.5 M NaCl followed by centrifugation (13,000× g, 30 min, 4°C). The pellets were dissolved in PBS containing 0.1% BSA (Sigma-Aldrich) in a tenth of the original culture volume and stored at 8°C.

### Phage display selection

Selections were performed in three rounds using streptavidin-coated paramagnetic Dynabeads (Dynal, Oslo, Norway). Every selection round was preceded by an incubation step, in which phages that bind non-specifically to the beads or that recognize the major substrate, i.e. xylan, of CBM4-2 were removed. In short, 500 μl of library phage-stocks were incubated in selection buffer (PBS, 1% BSA and 0.05% Tween 20) with 50 μl streptavidin-coated Dynabeads, i.e. beads without xyloglucan, and 400 μl xylan (soluble fraction of 100 mg xylan dissolved in 5 ml PBS). The beads were removed using a magnetic holder and the remaining supernatant was used for selections. In a parallel step Dynabeads were coated with biotin-conjugated xyloglucan. Different amounts of beads and xyloglucan were used in the different rounds of selection. In the first round 50 μl beads were coated with 2 μg biotin-labelled xyloglucan for 30 minutes before excess xyloglucan was removed by washing. The washed xyloglucan-coated beads were mixed with the supernatants from the preincubation step and left for incubation during 2 hours on an end-to-end rotor. Next, unbound phages were removed by washing four times with selection buffer and then twice with PBS. The bound phages were eluted by digestion with 100 μl of 0.5 mg/ml trypsin during 30 min after which the reaction was stopped by addition of 100 μl of 0.1 mg/ml aprotinin. Finally the eluted phages were rescued by infecting them into *E. coli *Top10F' at exponential growth phase during 30 min at 37°C. Bacteria were spread on selective media (LB-agar plates, 100 μg/ml ampicillin, 10 μg/ml tetracycline and 1% (w/v) glucose) and grown over night at 37°C. The second round of selections differed by reduction of the amount of ligand to 5 μl Dynabeads coated with 0.01 μg xyloglucan. In the third round of selections two different strategies were used. First, after preincubation of phage stocks with streptavidin-coated Dynabeads and soluble xylan, 0.01 μg biotin-xyloglucan was added to the phage stock. After a 30 minutes incubation at room temperature, 5 μl streptavidin-coated Dynabeads were added with or without concomitant addition of soluble xyloglucan (1.4 μg) as a competitor. The third round of selections generated four pools of phages (two each from Lib1 and Lib2) from which 40 colonies (10 from each pool) were picked at random and analyzed by phage ELISA and by sequencing. Isolated clones were named XG-34 followed by an Arabic number to define its library origin (Lib1 or Lib2) and a roman numeral to define the clone number (selected with (I-X) or without (XI-XX) added soluble xyloglucan during the final stages in the third round of selection).

### Phage ELISA

Binding of phage-displayed CBM towards xyloglucan and xylan was studied by ELISA. 96-well microtitre plates (Nunc, Roskilde, Denmark) were coated over night with strepavidin (0.2 μg/ml) or xylan (100 μl of the soluble fraction of 100 mg xylan dissolved in 11 ml PBS). Biotinylated xyloglucan (0.2 μg/ml) was added onto the streptavidin-coated plates. Phage stocks diluted in selection buffer were added to the plates in duplicates. After incubation for 2 hours at 37°C unbound phages were washed off and bound phages were detected using horseradish peroxidase-conjugated anti-M13 antibody (Amersham Pharmacia Biotech Inc., Piscataway, NJ, USA) diluted in blocking buffer. The plates were analyzed with *o*-phenylenediamine as chromogen whose development was quantified using an Emax spectrophotometer (Molecular Devices, Sunnyvale, CA, USA) at 490 nm, once the reaction had been stopped with H_2_SO_4_. In primary screening of clones, those that gave an absorbance > 0.2 against xyloglucan were considered to display XGBM.

### Sequence analysis

Sequencing was performed by Eurofins MWG Operon (Ebersberg, Germany) using plasmids purified from over night cultures using QIAamp DNA Mini Kit (Qiagen) as the sequencing template. Gene sequences have been deposited in GenBank [GenBank:FJ556578 (XG-34/1-X); GenBank:FJ556579 (XG-34/2-I); GenBank:FJ556580 (XG-34/2-VI)]. A structure model of XG-34/I-X was obtained using the CPHmodels 2.0 homology-modelling server http://www.cbs.dtu.dk/services/CPHmodels/ using the structure of CBM4-2 [PDB:1k42] as template, as described by Cicortas Gunnarsson *et al *[15].

### Site-directed mutagenesis

Single mutations in the XG-34 and XG-34/2-VI genes were introduced using QuickChange II Site-Directed Mutagenesis Kit (Stratagene) according to the manufacturer's protocol with one exception. Instead of using NZY Broth when recovering transformed *E. coli *XL1-Blue cells 2YT was used. To mutate XG-34 aspartate at position 112 into glutamate, primers 5'-GAATCAGTCGCATGATGAATACGGGAGACTGCATG-3' (forward) and 5'-CATGCAGTCTCCCGTATTCATCATGCGACTGATTC-3' (backward) were used. In the same way a primer-pair consisting of 5'-GAATCAGTCGCATGATGATTACGGGAGACTGCATG-3' (forward) and 5'-CATGCAGTCTCCCGTAATCATCATGCGACTGATTC-3' (backward) were used for mutation of the same residue (112) from glutamate to aspartate in XG-34/2-VI. Primers were obtained from Eurofins MWG Operon.

### Protein production

Production of soluble CBM was performed using the T7 expression-system consisting of the pET22b vector (Novagen, Madison, WI) harboured in *E. coli *BL21 (DE3). Restriction-sites were introduced before and after CBM-encoding genes with the help of PCR according to Cicortas Gunnarsson *et al *[6]. Digestion of purified PCR-products and pET22b with *Nde*I and *Xho*I (both from New England Biolabs) enabled cloning of the genes in-between *Nde*I/*Xho*I sites in the vector resulting in constructs encoding recombinant proteins containing a hexahistidine tag. The ligated products were transformed into chemically competent *E. coli *BL21 (DE3) from which production of recombinant CBM was controlled with glucose/IPTG. CBM-producing bacteria were cultured in 2YT media containing ampicillin (100 μg/ml) to exponential phase (OD_600 _= 0.3-0.5) before protein-production was induced by addition of IPTG (0.25 mM final concentration) and allowed to proceed for 3 hours at 37°C. Cells were harvested by centrifugation (6000× g, 15 min), washed with cold 0.9% NaCl and resuspended in 20 mM NaH_2_PO_4_/0.75 M NaCl, pH 7.4. Cell-lysates were centrifugated (13,000× g, 15 min) after sonication (3 × 2 min, 50% amplitude (250-D sonifier; Branson Ultrasonics Corp., Danbury, CT, USA)) and the CBM were finally purified on columns containing Ni-NTA (Qiagen) using immobilized metal affinity chromatography. The concentration of purified CBM was determined spectrophotometrically from absorbance measurements at 280 nm using extinction coefficients individually calculated for each CBM using the ProtParam software [16] available at http://www.expasy.org.

### Binding analysis

Affinity electrophoresis (AE) was done using polyacrylamide gels containing different carbohydrates. The gels used for separation were cast from carbohydrate-solutions containing 12.5% acrylamide/bisacrylamide (29:1) in 0.75 M Tris, pH 8.8. On top of each separation gel a stacking gel (3% acrylamide/bisacrylamide (29:1), 0.25 M Tris, pH 6.8) was cast. All gels were run using the Bio-Rad (Hercules, CA, USA) mini-gel system at room temperature at 90 V in running buffer (0.025 M Tris/0.192 M glycine, pH 8.3). The migration speed of native soluble CBM was performed using 3 μg protein per lane. The carbohydrates embedded in the gels were xyloglucan (0.125-2 mg/ml), birchwood xylan (0.125-2 mg/ml), laminarin (1 mg/ml) and lichenan (0.5 mg/ml). The proteins were visualized by SimplyBlue-staining (Invitrogen). A standard Kaleidoscopic protein-ladder (Bio-Rad) was included in the analysis as well as two earlier described CBM that served as controls, X-2 [15] that recognizes xylan but not xyloglucan, and G-4 [17] that does not bind to carbohydrates.

Competitive ELISA was performed to study how binding of CBM to immobilized xyloglucan was inhibited by pre-incubation with (biotinylated) xyloglucan and fucosylated xyloglucan. The inhibitory concentration required to reduce the signal by 50% (IC_50_) for the different variants was calculated. Flat-bottom 96-well plates (Nunc) were coated with 100 μl 50 nM xyloglucan over night at 4°C. XG-34 and its evolved variants were incubated with different concentrations of xyloglucan or fucosylated xyloglucan dissolved in PBS, 1% BSA, 0.05% Tween 20 during 1 hour at 37°C prior to transfer of the CBM-carbohydrate solutions to the xyloglucan-coated plates. Detection of CBM bound to immobilized xyloglucan was achieved by using horseradish peroxidase-conjugated anti-His_6 _antibody (Roche Diagnostic Corporation, Indianapolis, IN). Development of the plates was done as described above for phage ELISA.

Isothermal titration calorimetry (ITC) measurements were performed at 25°C following standard procedures [18] using a Microcal Omega titration calorimeter (Northampton, MA, USA). XXXG and XLLG (Figure 1) were prepared as previously described [19]. Prior to analysis, the CBM were extensively dialyzed against 20 mM HEPES, pH 8.0 containing 2 mM CaCl_2_. The same buffer was used to dissolve tamarind seed xyloglucan, XXXG, XLLG and galactose to minimize heats of dilution. 50 μM protein samples were stirred at 300 rpm in a 1.4331 ml reaction cell. Ligands were injected as a single 1 μl aliquot followed by 27 successive 10 μl aliquots at 300 s intervals (2 mM, 7.5 mM or 10 mM XXXG; 1 mM or 5 mM XLLG; 2.5% or 5% w/v xyloglucan; 10 mM galactose). Integrated heat effects were analyzed by non-linear regression using a single-site binding model.

### Plant tissue labelling with CBM and antibodies

Dry tamarind seeds were scarified and left overnight in water at room temperature (rt). The next day, swollen seeds were shelled and cut into ca 15 μm sections using a Leitz microtome (Leica Mikrosystems GmbH, Wetzlar, Germany) microtome. All labelling experiments were thereafter performed three times with sections placed in Eppendorf tubes. In order to prevent non-specific binding of CBM, sections were treated with a blocking solution containing 5% (w/v) ovalbumin in 50 mM sodium phosphate buffer (pH 7.4) for 1 h at rt followed by washing (twice, 15 minutes) in the same buffer. Tamarind sections were incubated with FITC-conjugated [20] CBM at 0.1-10 μM for 1 and 4 h (at rt) and overnight (+4°C). Thereafter samples were washed three times with buffer (10 minutes each step). Additional sections were labelled with fucosylated xyloglucan-specific FITC-conjugated CBM FXG-14b (Cicortas Gunnarsson *et al*, unpublished data) at a concentration of 2 μM in 50 mM sodium phosphate buffer (pH 7.4) using the same labelling protocol.

Blocked tamarind sections were also incubated with monoclonal antibody CCRC-M1 [13] at a 1:10 dilution in 50 mM sodium phosphate buffer (pH 7.4) supplemented with 1% ovalbumin overnight (+4°C). After washing (twice, 20 minutes each, rt with slight stirring), samples were labelled with FITC-conjugated anti-mouse antibody (Sigma-Aldrich) at a 1:500 dilution for 1 h at rt and washed with water.

### Fluorescent microscopy

Samples were placed on object glasses mounted in Fluorsave (Calbiochem), covered by coverslips and examined by fluorescent microscopy using a standard set of filters for FITC. All images were obtained using a Leica DC300F CCD camera and digital imaging system for professional microscopy (Leica Microsystems GmbH) at equal settings (magnification ×40, exposure time 1 s and gain 3.2).

## Results

### Library construction

**Figure 2:**
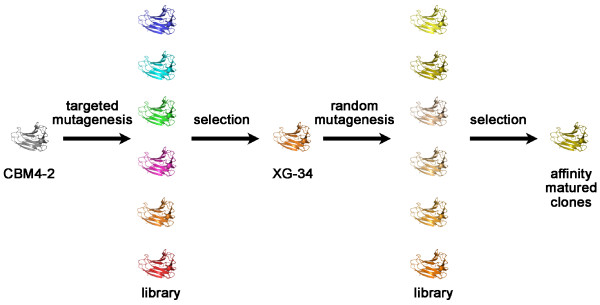
**CBM selection and evolution scheme**. Scheme showing the route of CBM evolution. Targeted mutagenesis of 12 codons encoding the carbohydrate-binding cleft of CBM4-2 resulted in a first library displayed on phage [6] from which the XGBM XG-34 was isolated [14]. Random PCR-based mutagenesis of the gene encoding XG-34 resulted in second generation phage display libraries from which matured variants of XG-34 could be selected by a stringent selection procedure (this investigation).

We have previously reported on the molecular engineering of xyloglucan-specific binders using the scaffold of a carbohydrate-binding module CBM4-2 from the xylanase 10A of *R. marinus*. In this study we show that it is possible to evolve (Figure 2) the functionality of binders based on this scaffold as exemplified by one such module, XG-34 specific for non-fucosylated xyloglucan [14] while preserving its specificity for xyloglucan. Through random mutagenesis, two libraries (Lib1 and Lib2) each consisting of 1-3 × 10^7 ^clones were created by cloning of mutated genes in a modified version of the pFAB5c.His phagemid vector [6] that is suited for use in phage display selection approaches. Sequencing of 23 clones randomly selected from each library revealed that 60% and 35% respectively, of members of Lib1 and Lib2 had inserts, out of which approximately 86% and 54% respectively, were in-frame. The frequency of base pair substitutions was 0.8-1.0%.

### Selection and primary screening

**Figure 3:**
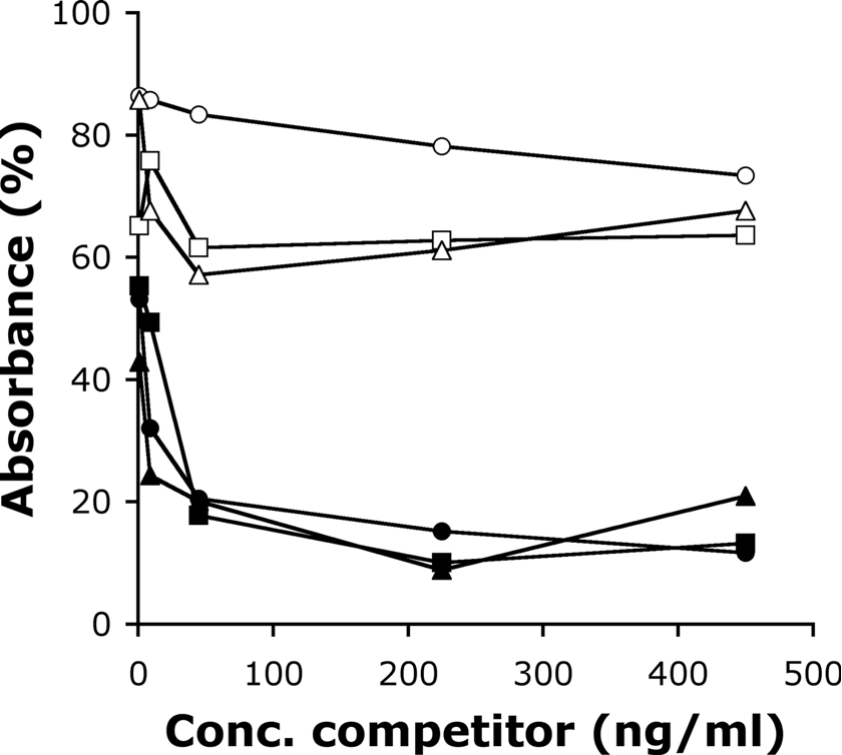
**Specific binding**. Binding of individual clones by phage-ELISA. Binding to immobilised biotin-labeled xyloglucan was inhibited by pre-incubation of XGBM-displaying phage clones with either soluble xyloglucan or xylan. The binding of three clones, XG-34/1-X (squares), XG-34/2-I (triangles) and XG-34/2-VI (circles), was inhibited by xyloglucan (filled symbols) but not by xylan (open symbols).

The combinatorial libraries were used separately for selection of tight xyloglucan-binding clones by phage display. Three rounds of selections were performed in the presence of xylan using biotinylated xyloglucan bound to streptavidin-coated Dynabeads as the target. The selection pressure was increased in two subsequent rounds of selection by decreasing the amount of biotinylated xyloglucan. In the last round of selection preformed biotinylated xyloglucan-phage-complexes were caught on streptavidin coated paramagnetic beads in the presence or absence of soluble xyloglucan in an attempt to vary the degree of selection stringency further. The selection system generated two pools of clones for each library. From each pool, 10 clones were randomly picked and analyzed with phage-ELISA using xylan or xyloglucan as target. Of these 40 clones, 31 bound xyloglucan. Among these, the six clones that bound best to xyloglucan in comparison to XG-34 and only showed low binding to xylan were selected. Subsequent analysis of the ability of soluble xyloglucan to inhibit the binding of these XGBM displayed on phage to immobilized xyloglucan, identified three clones with improved properties (Figure 3). These variants, XG-34/1-X (selected from Lib1) and XG-34/2-I and XG-34/2-VI (selected from Lib2), were all identified from the selections in which soluble xyloglucan had been incorporated in the final stages of the third round of selection.

### Affinity and specificity

**Figure 4:**
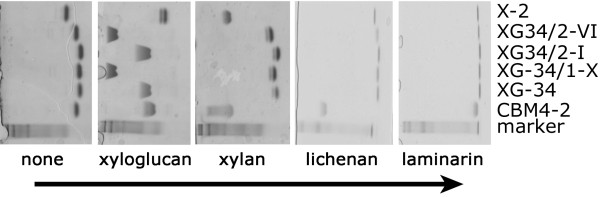
**Carbohydrate binding specificity**. AE in polyacrylamide gels polymerized in the presence of no substrate, 0.5 mg xyloglucan/ml, 1.0 mg xylan/ml, 0.5 mg lichenan/ml or 1.0 mg laminarin/ml. The arrow indicates the direction of migration in the gels. The evolved modules XG-34/1-X and XG-34/2-VI exhibited efficient binding to xyloglucan while XG-34/2-I bound slightly better than XG-34 and CBM4-2. The evolved clones do not show affinity for the other polysaccharides indicating that the specificity for xyloglucan was retained during the affinity maturation process. The first lane contains a kaleidoscopic protein-ladder to monitor gel migration and in the last lane an evolved xylan-specific CBM, X-2 [15] serves as a control.

The binding characteristics of the three variants of XG-34 in soluble form were analyzed by AE. Retardation in xyloglucan-containing gels was considerably higher for two of the evolvants, XG-34/1-X and XG-34/2-VI, than for XG-34 indicating improved affinity for these modules. The third mutant, XG-34/2-I behaved similar to XG-34. To assess the specificity of the new clones we performed AE-studies with laminarin, lichenan and different concentrations of xylan. None of the novel clones bound to any of these substrates demonstrating that polyreactive binding properties had not developed as a consequence of the affinity maturation process (Figure 4).

Binding affinities exhibited by XG-34, XG-34/1-X and XG-34/2-VI towards xyloglucan and the component oligosacharides XXXG (non-galactosylated) and XLLG (galactose-containing) (Figure 1) were determined by ITC. For XG-34, binding was clearly detectable but too low to be precisely quantified. In contrast, the evolvants XG-34/1-X and XG-34/2-VI bound well to XLLG in an enthalpy-driven interaction, with affinities in the K_A _= 10^5 ^M^-1 ^range, but not to the non-galactosylated oligosaccharide XXXG (Table 1). These modules also bound notably stronger to xyloglucan than XG-34, however still below the limits of precise quantification by ITC. The fact that these modules bind well to XLLG but not to XXXG suggests that the galactose decorations of the xyloglucan polymer play an important role in the binding of the module to the ligand. Further ITC experiments showed that neither XG-34/1-X nor XG-34/2-VI bind to galactose monosaccharides, strongly suggesting that a more specific, ordered pattern of the galactose decorations provide a necessary motif for proper ligand recognition and binding.

**Table 1:** Binding affinity constants (K_A_) and thermodynamic properties of XGBM-carbohydrate interactions*.

Module	Carbohydrate							
	
	xyloglucan	XXXG	XLLG^†^					galactose
				
	*K*_A_ (10^3 ^M^-1^)	*K*_A_ (10^3 ^M^-1^)	*K*_A_ (10^3 ^M^-1^)	*ΔG* (kcal mol^-1^)	*ΔH* (kcal mol^-1^)	*TΔS* (kcal mol^-1^)	n^§^	
XG-34	≈ 0.1	≈ 1	≈ 1	-	-	-	-	not determined
XG-34/1-X	≈ 10	≈ 1	147 ± 6	-7.0 ± 0.1	-15.8 ± 0.1	-8.8 ± 0.1	1.0 ± 0.0	no binding
XG-34/2-VI	≈ 10	≈ 1	141 ± 4	-7.0 ± 0.0	-14.0 ± 0.1	-7.0 ± 0.1	1.1 ± 0.0	no binding

*Thermodynamic parameters for some interactions could not be fully characterized due to relatively low affinity, limited solubility of protein and limited availability of ligand. In those cases n was fixed to 1 for data fitting and calculation of *K*_A_.

^†^ITC thermograms are available as supplementary information [see Additional file 1]

^§^calculated number of binding sites on the protein

**Figure 5:**
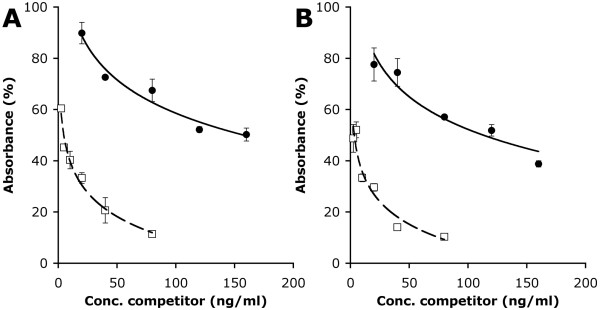
**Carbohydrate binding specificity**. Inhibition of binding of XG-34/1-X (A) and XG-34/2-VI (B) to immobilized xyloglucan by either soluble fucosylated xyloglucan (filled circles) or non-fucosylated xyloglucan (empty squares).

To further define the specificity of the two tight-binding mutants we investigated if these possessed, like XG-34, the ability to discriminate xyloglucan from its fucosylated form. Competition ELISA was performed by inhibiting binding of XG-34/1-X and XG-34/2-VI to biotinylated xyloglucan on plates by preincubation with different concentrations of soluble (biotinylated) xyloglucan or fucosylated xyloglucan. XG-34/1-X and XG-34/2-VI were efficiently inhibited from binding immobilized xyloglucan by addition of soluble xyloglucan with an IC_50_-value of 5 ng/ml and 3 ng/ml, respectively. To achieve the same degree of inhibition approximately 30-fold more fucosylated xyloglucan was needed (Figure 5), revealing that the ability of XG-34 to discriminate between these forms of xyloglucan had been retained during the affinity maturation process.

### Sequence analysis

**Figure 6:**
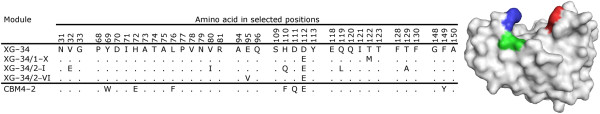
**Protein sequences**. Sequence alignment of XG-34, the three evolved modules XG-34/1-X, XG-34/2-I and XG-34/2-VI and CBM4-2. Residue numbering is in agreement with Simpson *et al *[22]. Dots indicate identity with XG-34 sequence. The close proximity of residue 112 (green), mutated in evolved variants of XG-34, to residues 110 (blue) and 69 (red) that are important for carbohydrate binding in proteins based on the scaffold [15,22], is highlighted on a structure model of XG-34/1-X.

Sequencing revealed that XG-34/1-X, XG-34/2-I and XG-34/2-VI carried 2-6 substitutions at the amino acid level in comparison to XG-34. They had one mutation in common, a substitution of aspartate in position 112 to glutamate (Asp^112 ^→ Glu). This substitution reverses a mutation of XG-34 to the residue found in CBM4-2 (Figure 6). Besides this mutation XG-34/1-X and XG-34/2-VI, the two tight-binding clones, had only one other mutation each, both of which were located in two adjacent loops far away from the carbohydrate binding-site. The number of substitutions found in the third clone, XG-34/2-I was greater as it had incorporated 6 altered residues (Figure 6).

### Mutagenesis of residue 112

**Figure 7:**
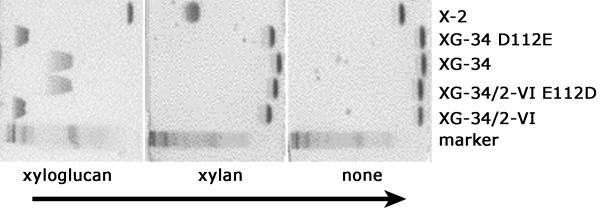
**Asp/Glu^112 ^and binding affinity**. AE of XGBM XG-34/2-VI and Asp^112 ^→ Glu-mutated XG-34 that both contain glutamate in position 112 demonstrate that they bind stronger to xyloglucan than their aspartate-containing counterparts. None of these modules show binding to xylan that is recognized by the xylan-specific module X-2 [15]. The arrow indicates the direction of migration in the gels.

To evaluate the significance of glutamate in position 112, a Glu^112 ^→ Asp mutant of XG-34/2-VI and an Asp^112 ^→ Glu mutant of XG-34 were produced. Affinity electrophoresis demonstrated that XGBM containing glutamate in position 112 i.e. XG-34/2-VI and XG-34 D112E, bound xyloglucan with high affinity. In contrast, proteins having an aspartate in position 112 (XG-34 and XG-34/2-VI E112D) bound xyloglucan with low affinity (Figure 7). Thus, the glutamate in position 112 alone is responsible for the increased apparent affinity of XG-34/2-VI for xyloglucan and it is likely to have a significant effect on the affinity of XG-34/1-X and XG-34/2-I as well. As none of these proteins with aspartate or glutamate in this position, in contrast to CBM4-2, show any binding to xylan, it appears that other common differences (in positions 69, 72, 76, 110, 111, 149) in the sequence of XG-34 and its mutants in comparison to CBM4-2 are likely to contribute to this binding character. Thus, these other modifications provide the framework in which Glu^112 ^is able to mediate specific high affinity binding to xyloglucan.

### Detection of xyloglucan in tamarind seeds

**Figure 8:**
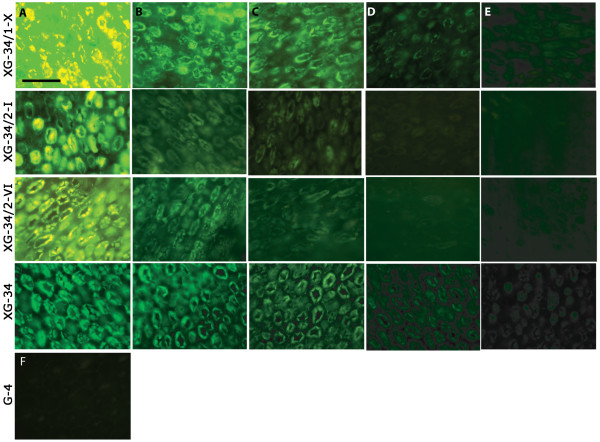
**Tamarind seed xyloglucan binding**. Staining of tamarind seed tissue sections using fluorescein-labeled XGBM as probes. The probes were used at 200 (A), 40 (B), 20 (C), 4 (D) and 2 (E) μg/ml. F illustrates the lack of staining with a protein-specific module that does not bind plant carbohydrates, G-4 [17] developed from the CBM4-2 scaffold, at 200 μg/ml. All sections were scanned with the same settings resulting in oversaturation of the signal for the best XGBM at the highest concentrations. Bar lines = 200 μm

**Figure 9:**
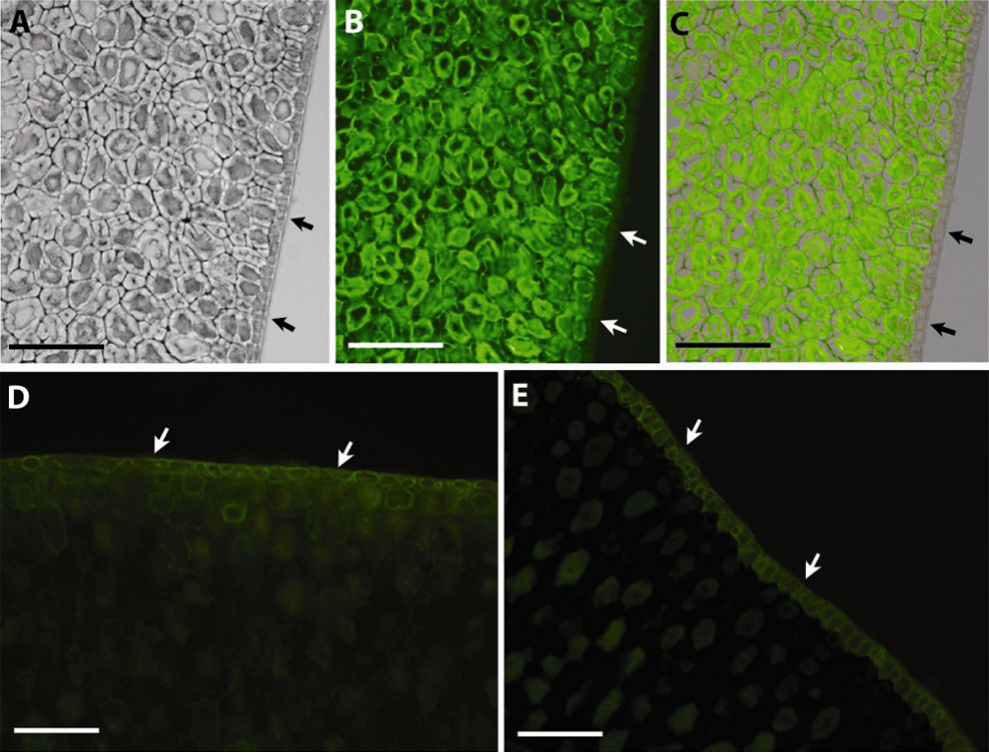
**Tamarind seed endosperm specificity**. Specificity of staining as illustrated by the inability of XGBM XG-34/1-X to stain tamarind seed integument (arrows) that is rich in fucosylated xyloglucan. Images show a section seen under visible light (A), the fluorescence of the same section (B) and a merged image thereof (C). While the module stained endosperm cells, the integument cells covering endosperm remained unstained. As a control, integument cells stained well with the monoclonal antibody CCRC-M1 (D) and the CBM FXG-14b (E) that are both specific for fucosylated xyloglucan. XGBM XG-34, XG-34/2-I and XG-34/2-VI all showed a staining pattern similar to that of XG-34/1-X (data not shown). Bar lines = 200 μm

An improvement in binding to a target of interest can potentially translate into improved performance of the probe in various applications. We explored how these xyloglucan-specific variants derived from the CBM4-2 scaffold behaved in the staining of endosperm of tamarind seeds, a source rich in non-fucosylated xyloglucan. As demonstrated in Figure 8, the evolution process had succeeded in creating variants that demonstrated more intense staining in an immunofluorescence application. In addition, the time of labelling did not affect signal intensity (i.e. after 1 h it was similar to 4 h and overnight labelling) suggesting a rapid and specific binding to non-fucosylated xyloglucan. Staining intensity of the CBM was also stable over at least 1 week after storage at 4°C. Importantly, the specificity of the modules for non-fucosylated xyloglucan could be confirmed as the tamarind seed integument rich in fucosylated but not non-fucosylated xyloglucan, was not stained by XG-34 or by modules evolved from it (Figure 9A-C). In contrast, other reagents specific for fucosylated xyloglucan stained the tamarind seed integument but not the endosperm (Figure 9D-E)

## Discussion

In the search for analytical probes we have focused on investigating the potential of CBM4-2 as an alternative to the antibody scaffold for molecular engineering of modules with novel binding characteristics. We have in previous studies created a combinatorial library on the CBM4-2 scaffold [6] from which modules that specifically bind different plant polysaccharides have been selected, e.g. XG-34 that recognizes only non-fucosylated xyloglucan [14]. In the present study we show for the first time that modules with the CBM4-2 scaffold can be further evolved using random mutagenesis in order to gain new binding properties such as improved affinity. This indicates a plasticity of the CBM4-2 scaffold and demonstrates its capacity for forced evolution.

The study resulted in the generation of three new xyloglucan-binding modules, XG-34/1-X, XG-34/2-I and XG-34/2-VI. Compared to existing xyloglucan probes such as monoclonal antibodies LM15 [12] that primarily binds to the non-galactosylated xyloglucan unit XXXG, and CCRC-M1 [13] that recognizes only fucosylated xyloglucan, our high affinity binders differ by recognizing the galactose-decorated xyloglucan unit XLLG, and by discriminating non-fucosylated from fucosylated xyloglucan. Unfortunately, other xylogluco-oligosaccharides like XLXG and XXLG that may be present in non-fucosylated xyloglucan could not be assessed by ITC as they were not available in sufficient quantities in pure form preventing a more complete specificity analysis. Nevertheless, the combined data demonstrates a specificity of these modules different from the other existing probes. Besides having higher affinity than XG-34 for soluble xyloglucan, the new binders also stained plant tissues from tamarind seed more intensely at high protein concentration and were at least equivalent at lower concentrations. These qualities demonstrate that the evolution achieved a molecular character that translated into an improved performance in terms of staining intensity in an analytical setting. The enhanced labelling may be a consequence of a higher affinity of the evolved variants not only for soluble xyloglucan, as assessed by ITC, but also for some xyloglucan structures in these samples. More specifically, enhanced staining may be a consequence of a slightly modified fine specificity increasing the number of structures recognized in the tissue. Indeed, molecular evolution of antibodies, the golden standard of binding probes, has been shown to be accompanied by such specificity fine-tuning [21]. Altogether, the improved xyloglucan-binding modules can readily be utilized for xyloglucan recognition in plant sections and due to their novel binding properties that differentiate them from other xyloglucan probes, they provide a novel set of analytical tools, complementary to the existing ones.

One feature of the evolved xyloglucan binders is that all contained one mutation in common (aspartate to glutamate in position 112) compared to XG-34. This mutation was shown to be crucial for the gained affinity for one of the modules. Unfortunately, attempts to obtain crystal complexes of the CBM evolvants with xylogluco-oligosaccharides have so far been unsuccessful. Consequently, the detailed structural role of the critical residue 112 in xyloglucan recognition is not fully understood. Residue 112 is however most likely a residue that directly contacts carbohydrate ligands. In the wild type CBM4-2 and in models of evolved variants of XG-34, the side chain of this residue is oriented towards the cavity into which carbohydrates are likely to bind [22] (Figure 6) [see Additional file 1] and NMR signals from the backbone amide of residue 112 shifts upon addition of either xylopentaose or cellohexaose [15] suggesting that it is involved in ligand binding. Similarly, the side chain of this residue has been shown to be located in immediate proximity to the ligand in recently solved structures of a xylan-specific variant in complex with oligoxylose (von Schantz *et al*, unpublished data). It thus appears that the evolution process targeted a residue directly involved in ligand interaction. This is in contrast to antibodies whose evolution has often shown to result from mutations in the periphery or outside the binding-cleft both *in vivo *and *in vitro *[23-26]. Future evolution studies will have to confirm whether this is a general evolutionary pathway for variants originating from the CBM4-2 scaffold or if mutations distant from the binding-cleft also are, as they are for antibodies, capable of fine-tuning ligand-binder affinity. Glu^112 ^in combination with other mutations present in XG-34/2-I (Val^32 ^→ Glu, Val^80 ^→ Ile, His^110 ^→ Gln, Gln^119 ^→ Leu, Thr^129 ^→ Ala) did not however result in an improved affinity, suggesting that one or several of these other mutations counteract the contribution in affinity for xyloglucan promoted by Glu112. One of the mutated residues, 110, is a critical residue in the binding site of CBM4-2 [22]. This modification may thus be a major contributor to the relatively poor affinity of XG-34/2-I despite the presence of the Asp^112 ^→ Glu modification in this module.

The selection strategy involving capturing of biotinylated xyloglucan-phage complexes in the presence of soluble xyloglucan was successful as all three evolved CBM were extracted from the libraries by applying this methodology. Thus, stringent conditions appeared to be required to find specific XGBM variants with higher affinity from libraries created from the CBM4-2 scaffold. This is in concordance with antibody phage methodology where it is known that binders with different characteristics can be identified by choosing different selection conditions [27]. Thus, the same rules appear to apply to the selection of specific binders derived from the CBM4-2 scaffold, a fact that can be exploited to find binders optimal for a given application.

## Conclusion

In conclusion, our study shows that the binding characteristics, such as binding affinity, of modules selected from molecular libraries created on the CBM4-2 scaffold can be further improved by a molecular evolution process. This evolution resulted in the creation of three novel xyloglucan binders, out of which two showed greatly improved affinity for xyloglucan. The variants have a defined specificity, as they could be shown to specifically target galactose-decorated xyloglucan structures (such as XLLG). In this respect, the two high affinity modules XG-34/1-X and XG-34/2-VI differ substantially from existing xyloglucan binders and constitute a novel analytical instrument for xyloglucan detection in plant sections. The great flexibility that can be applied to selection from phage-display libraries provides the scientist a substantial advantage over conventional monoclonal antibody technology in the search for binders with selected and defined properties. We envisage that the use of these technologies will provide a wide repertoire of defined reagents, important for the detailed analysis of plant tissues and plant-derived industrial material in the future.

## List of abbreviations used

AE: affinity electrophoresis; CBM: carbohydrate binding module; IC_50_: inhibitory concentration required to reduce the signal by 50%; ITC: isothermal titration calorimetry; K_A_: affinity constant; rt: room temperature; XGBM: xyloglucan binding module.

## Authors' contributions

LCG, ENK and MO designed the study; SS and LCG were responsible for library selection; LvS had the main responsibility for design and generation of mutants and most aspects of protein characterization; FG, HB and JEF designed and conducted ITC studies; GD and LF designed and conducted tissue section studies; LvS and MO drafted the manuscript. All authors critically revised and approved the manuscript.

## Supplementary Material

Additional file 1 **Supplementary information related to "Affinity maturation generates greatly improved xyloglucan-specific carbohydrate binding modules"**. The file show ITC thermograms used to determine affinity constants and illustrate a comparison of the structure of CBM4-2 with a model of XG-34/1-X. Click here for file
