# Supplementary material for: Affinity maturation generates greatly improved xyloglucan-specific carbohydrate binding modules
Source: BMC Biotechnol. 2009 Oct 31;9:92. doi: 10.1186/1472-6750-9-92 (PMC2783032; doi:10.1186/1472-6750-9-92)
Supplement: Additional file 1 — Supplementary information related to "Affinity maturation generates greatly improved xyloglucan-specific carbohydrate binding modules". The file show ITC thermograms used to determine affinity constants and illustrate a comparison of the structure of CBM4-2 with a model of XG-34/1-X. [file 1472-6750-9-92-S1.pdf]

Additional file 1

Supplementary information related to
Affinity maturation generates greatly improved xyloglucan-specific carbohydrate binding modules

Laura von Schantz[*], Fredrika Gullfot[†], Sebastian Scheer[*], Lada Filonova[‡,¶]
Lavinia Cicortas Gunnarsson[*], James E. Flint[§], Geoffrey Daniel[‡,¶], Eva Nordberg-Karlsson[||],
Harry Brumer[†], Mats Ohlin[*]

[*]Dept. of Immunotechnology, Lund University, Lund, Sweden; [†]School of Biotechnology, Royal Institute of Technology (KTH), Stockholm, Sweden; [‡]Dept. of Wood Science, Swedish University of Agricultural Science, Uppsala, Sweden; [¶]WURC, Swedish University of Agricultural Science, Uppsala, Sweden; [§]Institute for Cell and Molecular Biosciences, Newcastle University, Newcastle upon Tyne, UK; [||]Dept. of Biotechnology, Lund University, Lund, Sweden.

Content:

Page 2: Isotermal titration calorimetric thermograms describing the interaction of XG-34, XG-34/1-X and XG-34/2-VI with XLLG.

Page 3: Illustration of the structure of CBM4-2 and a model of XG-34/1-X with key residues highlighted.

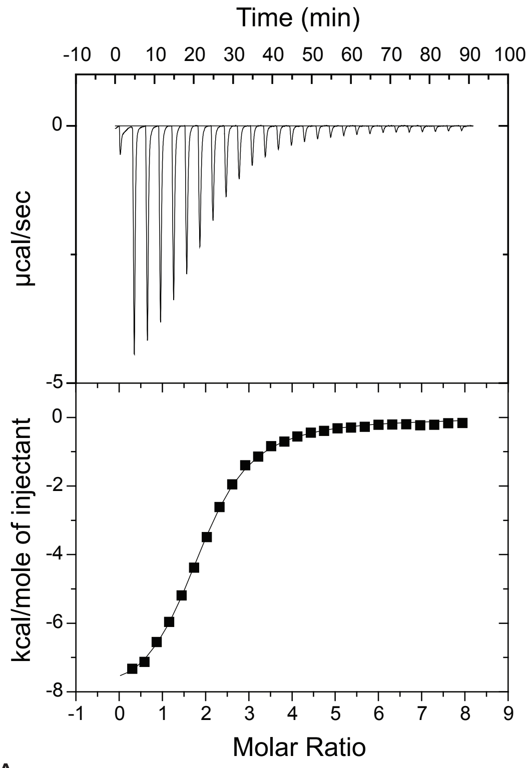

A

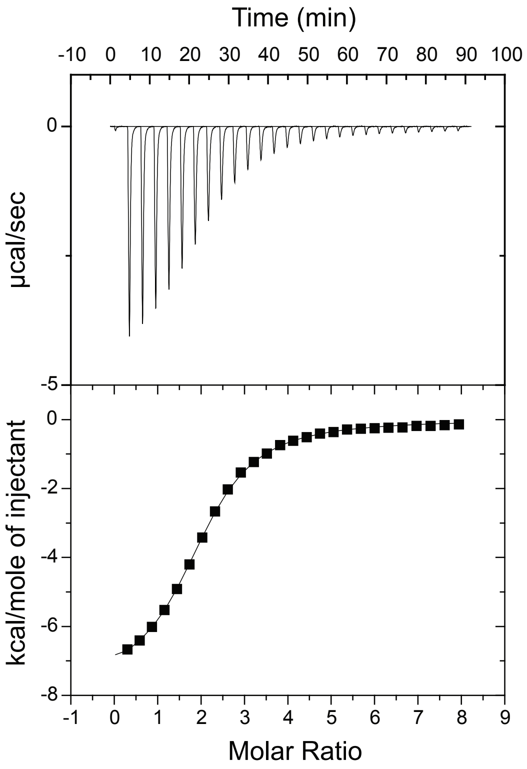

B

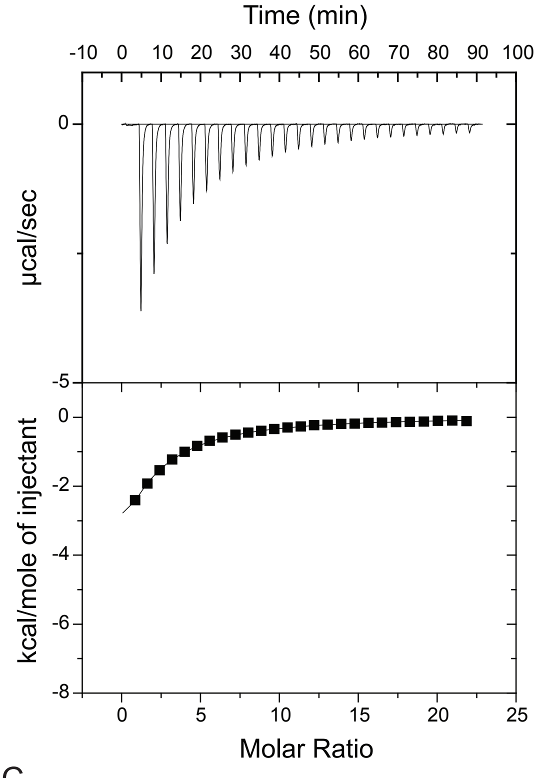

C

ITC thermograms with XLLG
A XG-34/1-X
B XG-34/2-VI
C XG-34

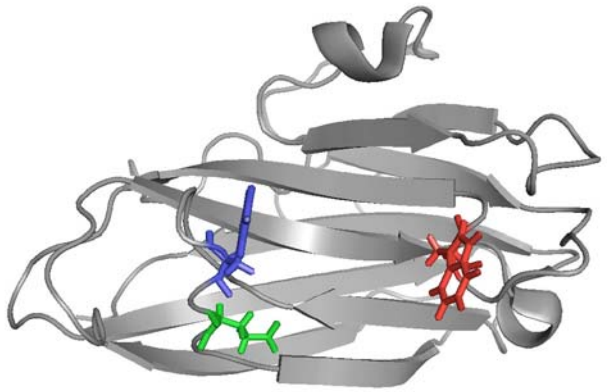

Structure of CBM4-2 (PDB: 1K45)

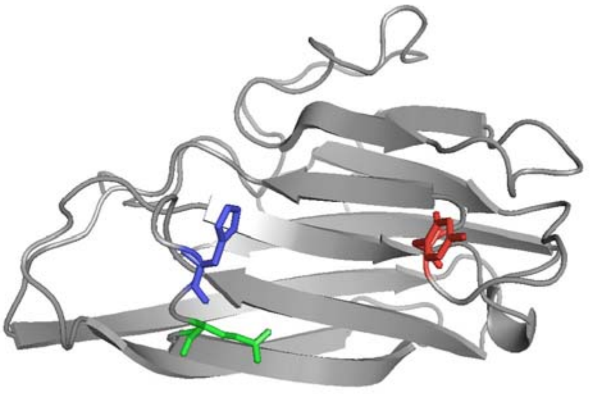

Structure model of XG-34/1-X

Illustration of the structure of CBM4-2 as determined by NMR (Simpson *et al.*, 2002) and the model of XG-34/1-X created by the CPHmodels 2.0 homology-modelling server (http://www.cbs.dtu.dk/services/CPHmodels/). The figures show top views of the respective binding sites with residue Glu^{112} associated to affinity maturation of xyloglucan binding highlighted in green. Key residues 69 and 110 are shown in red and blue, respectively.

Reference:

Simpson PJ, Jamieson, SJ, Abou-Hachem M, Karlsson EN, Gilbert HJ, Holst O, Williamson MP: **The solution structure of the CBM4-2 carbohydrate binding module from a thermostable Rhodothermus marinus xylanase.** Biochemistry 2002, **41:**5712-5719
